# Association Between Free Triiodothyronine and Metabolic Dysfunction-Associated Steatotic Liver Disease in Euthyroid Population: Evidence from NHANES 2007–2012

**DOI:** 10.3390/healthcare14213720

**Published:** 2026-11-02

**Authors:** Tianru Zhang, Shiyu Peng, Ping He, Xinke Wang, Baoping Nie, Yuemin Feng, Zhen Yu, Qiang Zhu

**Affiliations:** 1 Department of Gastroenterology, Shandong Provincial Hospital Affiliated to Shandong First Medical University, Jinan 250021, China; zhangtianru94@outlook.com (T.Z.); peacefulmia@163.com (P.H.); wangxinke_sdutcm@163.com (X.W.); niebaopingnie@163.com (B.N.); sdyueminfeng@163.com (Y.F.); 2 Department of Gastroenterology, The First Affiliated Hospital of Xinjiang Medical University, Urumqi 830054, China; medicalp123@163.com; 3 Department of Infectious Disease, Shandong Provincial Hospital Affiliated to Shandong First Medical University, Jinan 250021, China

**Keywords:** triiodothyronine, non-alcoholic fatty liver disease, fibrosis, nutrition surveys

## Abstract

**Highlights:**

**What are the main findings?**

- In euthyroid adults, higher FT3 was associated with higher MASLD risk; the inverse association with FIB-4-defined advanced fibrosis was exploratory and not robust after age adjustment.
- The FT4/FT3 ratio was inversely associated with MASLD in age-adjusted models; its association with advanced fibrosis was cross-sectional and came from a model without age adjustment.

**What are the implications of the main findings?**

- The fibrosis-related associations do not establish clinical utility.
- Exploratory unweighted analyses suggested that albumin and lymphocyte count were each associated with part of the FT3–MASLD association, but these findings do not establish causal mediation.

**Abstract:**

**Background/Objectives:** This study investigated the associations of free triiodothyronine (FT3) with metabolic dysfunction-associated steatotic liver disease (MASLD) risk and high probability of advanced liver fibrosis in euthyroid individuals. **Materials and Methods:** We conducted a cross-sectional analysis of 6910 participants from the 2007–2012 cycles of the National Health and Nutrition Examination Survey (NHANES). MASLD was defined using the non-invasive Fatty Liver Index (FLI), and high probability of advanced liver fibrosis was evaluated by the Fibrosis-4 (FIB-4) score. Weighted multivariable logistic regression and restricted cubic spline (RCS) analyses were used to assess associations between serum FT3 and the two hepatic outcomes. Exploratory, unweighted mediation analyses examined whether serum albumin (ALB) or lymphocyte count (LYM) statistically accounted for part of the FT3–MASLD association. These analyses are hypothesis-generating, and causal mediation cannot be inferred from this design. **Results:** Higher FT3 was positively associated with FLI-defined MASLD (per 1 SD, 0.385 pg/mL: OR = 1.34, 95% CI 1.18–1.53, *p* < 0.001). In the primary fibrosis model without age adjustment, lower FT3 was associated with high probability of advanced liver fibrosis (OR = 0.41, 95% CI 0.26–0.65, *p* < 0.001), but after continuous age adjustment, this association attenuated and became non-significant. The ratio of free thyroxine to free triiodothyronine (FT4/FT3 ratio) was inversely associated with MASLD; its positive association with high probability of advanced liver fibrosis (from an age-unadjusted model) remains exploratory. Exploratory analyses suggested ALB and LYM might partly account for the FT3–MASLD association. **Conclusions:** In euthyroid adults, higher FT3 and a lower FT4/FT3 ratio were cross-sectionally associated with increased MASLD risk. Associations of lower FT3 and a higher FT4/FT3 ratio with high probability of advanced liver fibrosis were exploratory, age-dependent, and not robust. The fibrosis-related findings are cross-sectional associations and do not establish clinical utility.

## 1. Introduction

Metabolic dysfunction-associated steatotic liver disease (MASLD) is a highly prevalent global metabolic disorder that is tightly intertwined with multiple systemic comorbidities, including obesity, type 2 diabetes mellitus, hypertension, sarcopenia, and cardiovascular disease (CVD) [1,2]. Formerly recognized as non-alcoholic fatty liver disease (NAFLD), MASLD was redefined through an international Delphi consensus to emphasize that MASLD is not merely an isolated hepatic disorder but a phenotypic manifestation of systemic metabolic dysregulation [3,4,5]. Epidemiological data indicate that the global prevalence of MASLD reached 32.4% in 2022 and continues to rise rapidly, establishing it as one of the most prevalent chronic liver diseases worldwide [6].

Thyroid hormones are pivotal regulators of systemic lipid and carbohydrate metabolism, and thyroid dysfunction has been well documented to be closely implicated in the occurrence and progression of chronic liver diseases [7,8,9,10,11]. A growing body of clinical and basic studies have indicated the involvement of thyroid hormone disorders in MASLD development and progression. In particular, primary hypothyroidism is significantly associated with both increased MASLD prevalence and aggravated histological severity [12]. A study using Mendelian randomization (MR) indicated that metabolic disorders mediate ∼41% of the hypothyroidism–MASLD association. These findings raise the possibility that variations in thyroid hormone concentrations, even within the reference range, may be associated with metabolic dysfunction. [13] Moreover, emerging mechanistic studies suggest that there is an ‘intrahepatic hypothyroid state’ in euthyroid patients with metabolic dysfunction-associated steatohepatitis (MASH), which is triggered by liver inflammation and fibrosis via hedgehog pathway activation, despite normal peripheral circulating thyroid hormone levels [14].

Given limited evidence on the association between free triiodothyronine (FT3) and the risk of MASLD in euthyroid population, this study explored the associations of normal range of FT3 with MASLD and high probability of advanced liver fibrosis, and further examined whether lymphocyte count (LYM) and serum albumin (ALB) might be associated with part of these relationships.

## 2. Materials and Methods

### 2.1. Study Population

The National Health and Nutrition Examination Survey (NHANES) is a series of national surveys assessing the health and nutritional status of adults and children in the United States. It is conducted by the National Center for Health Statistics (NCHS), part of the Centers for Disease Control and Prevention (CDC), to generate national health statistics. The NHANES protocol is approved by the NCHS Research Ethics Review Board, and all participants provide written informed consent.

**Figure 1:**
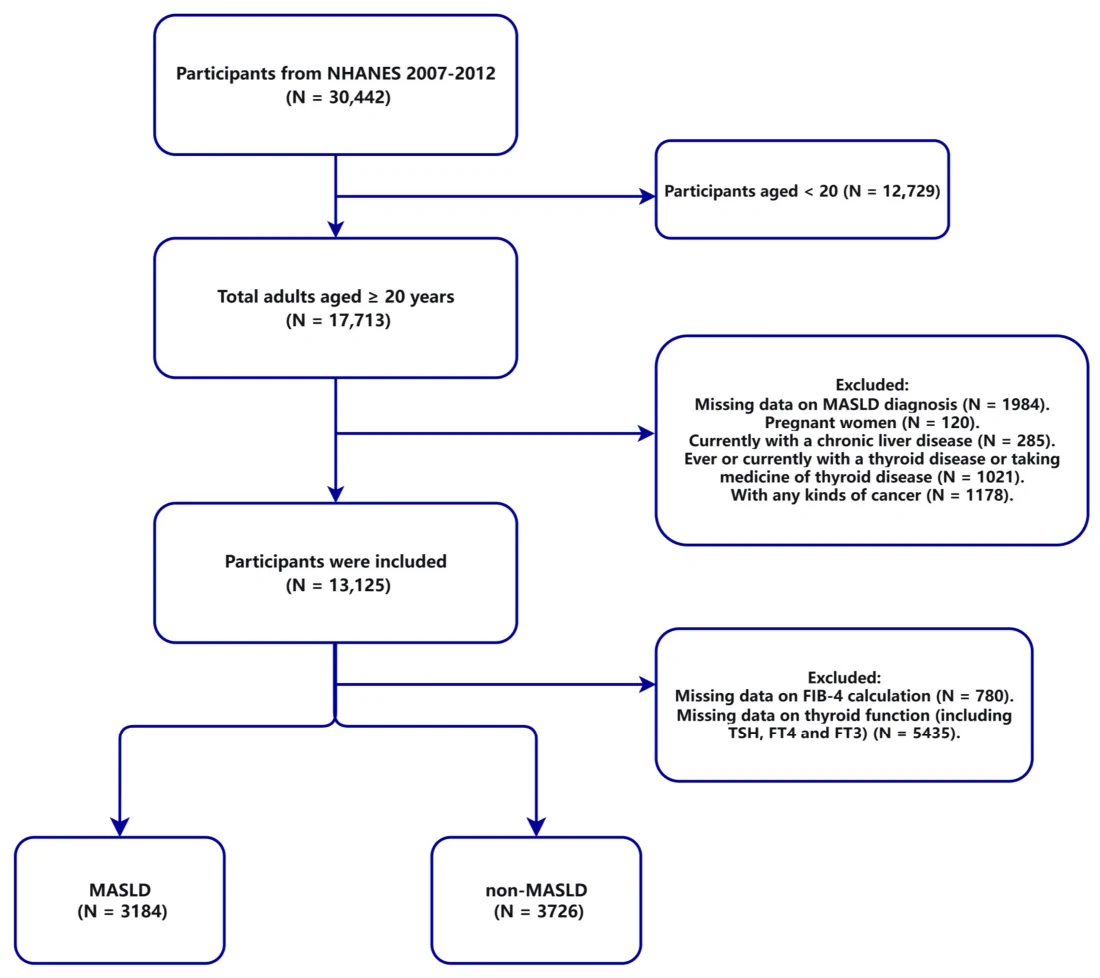
Flowchart of participant screening and enrollment.

In our investigation, a total of 30,442 participants were initially enrolled from three NHANES cycles (2007–2008, 2009–2010, 2011–2012). We first excluded individuals aged <20 years (N = 12,729), leaving 17,713 adults. Subsequently, we excluded pregnant participants (N = 120), those with pre-existing chronic liver disease (N = 285), those ever or currently with thyroid dysfunction or thyroid medication use (N = 1021), any malignancy (N = 1178), and those with missing data for MASLD diagnosis (N = 1984). Finally, we further excluded those with missing data on FIB-4 calculation (N = 780) and missing data on thyroid function (N = 5435). A final total of 6910 adult participants were included in this analysis, consisting of 3184 individuals with MASLD and 3726 non-MASLD controls. The detailed participant selection process is presented in Figure 1.

### 2.2. Thyroid Function

Serum FT3 and free thyroxine (FT4) levels were measured on a Beckman Coulter Access 2 Immunoassay System (Beckman Coulter, Inc., Brea, CA, USA) using chemiluminescent immunoassay. FT3 was quantified with a competitive binding immunoenzymatic assay, FT4 with a two-step enzyme immunoassay, and thyroid stimulating hormone (TSH) with a two-site immunoenzymatic (“sandwich”) technique, in accordance with the NHANES 2007–2012 laboratory protocol. In NHANES 2007–2012, reference intervals were 0.8–1.8 ng/dL for FT4, 2.0–4.4 pg/mL for FT3, and 0.4–4.0 mIU/L for TSH [15]. FT4-to-FT3 conversion can be assessed by the ratio of free thyroxine to free triiodothyronine (FT4/FT3 ratio), calculated as [FT4 (ng/dL) × 12.87]/[FT3 (pg/mL) × 1.536].

In the present study, euthyroid adults were defined as participants aged ≥20 years with the normal serum TSH reference range described above, and no self-reported history of thyroid disease or thyroid medication use. Individuals presenting isolated abnormal FT3 or FT4 values without clinical indications for thyroid treatment were still classified as euthyroid.

### 2.3. Covariates

Details regarding individuals’ sociodemographic factors, health behaviors, and health-related status were collected through questionnaire-based interviews and mobile examination centers (MECs). Sociodemographic factors included age (years), gender (male and female), race (Mexican American, non-Hispanic black, non-Hispanic white, other Hispanic), and marital (married/living with partner and widowed/divorced/separated/never married). A total of 510 participants who identified as “Other race (including multiracial)” were not included in the four predefined racial categories in Table 1; therefore, the sum of the racial categories is smaller than the total sample size. These participants were retained in the overall sample and in all other analyses (overall N = 6910). Health behaviors included drinking (yes, no). Alcohol consumption data were derived from the NHANES questionnaire. Missing values for this drinking (*n* = 502, 7.3%) were mainly due to questionnaire skip patterns.

Body mass index (BMI) was calculated as weight in kilograms divided by height in meters squared. Health-related variables comprised BMI, serum albumin (ALB, g/L), alanine aminotransferase (ALT, U/L), aspartate aminotransferase (AST, U/L), gamma-glutamyl transferase (GGT, U/L), alkaline phosphatase (ALP, U/L), and serum triglycerides (TG). Poverty income ratio (PIR) was included as a sociodemographic covariate. Diabetes was defined as the use of oral hypoglycemic agents, insulin, or a self-reported history of diabetes. Hypertriglyceridemia was defined as serum TG ≥ 1.7 mmol/L (150 mg/dL) or the use of lipid-lowering medication. A detailed description of the variables used in this research is available at https://www.cdc.gov/nchs/nhanes/ (accessed on 24 October 2026).

### 2.4. Outcome Definitions

Vibration-controlled transient elastography is widely used to assess hepatic steatosis and fibrosis. However, FibroScan measurements are not available in NHANES data prior to 2017. Therefore, we used the Fatty Liver Index (FLI) and the Fibrosis-4 (FIB-4) index to evaluate hepatic steatosis and high probability of advanced liver fibrosis, respectively.

The FLI, a non-invasive tool based on routine clinical parameters, was used to de-fine hepatic steatosis [16,17,18,19]. The FLI equation was as follows:

$$\mathrm{FLI}=\frac{e^{0.953\times \ln\left(\mathrm{TG}\right)+0.139\times \mathrm{BMI}+0.718\times \ln\left(\mathrm{GGT}\right)+0.053\times \mathrm{WC}-15.745}}{1+e^{0.953\times \ln\left(\mathrm{TG}\right)+0.139\times \mathrm{BMI}+0.718\times \ln\left(\mathrm{GGT}\right)+0.053\times \mathrm{WC}-15.745}}\times 100$$

where TG, BMI, GGT, and WC denote serum triglycerides, body mass index, gamma-glutamyl transferase, and waist circumference, respectively. An FLI ≥ 60 was used to define hepatic steatosis, which has been externally validated against ultrasound-proven steatosis, with a sensitivity of 61%, specificity of 86%, and positive likelihood ratio of 4.3 [16].

The FIB-4 index was used to assess the high probability of advanced liver fibrosis, particularly in individuals with chronic liver conditions such as hepatitis or metabolic-associated steatotic liver disease [20,21,22]. The FIB-4 equation was as follows:

$$\mathrm{FIB}\text{-}4=\frac{\mathrm{Age}\times \mathrm{AST}}{\mathrm{Platelet count}\times \sqrt{\mathrm{ALT}}}$$

FIB-4 ≥ 2.67 was used to identify participants with a high probability of advanced liver fibrosis [20,21,23].

In this study, hepatic steatosis was defined as FLI ≥ 60, and high probability of advanced liver fibrosis was defined as FIB-4 ≥ 2.67.

### 2.5. Definition of MASLD

MASLD was operationally defined as FLI-estimated hepatic steatosis (FLI ≥ 60) together with at least one cardiometabolic risk factor. The adult cardiometabolic criteria were:

(1) BMI ≥ 25 kg/m^2^ (23 kg/m^2^ for Asians) or WC > 94 cm in men and > 80 cm in women;
(2) Fasting blood glucose (FBG) ≥ 5.6 mmol/L (100 mg/dL) or 2 h post-load blood glucose ≥ 7.8 mmol/L (140 mg/dL) or glycated hemoglobin (HbA1c) ≥ 5.7% (39 mmol/mol) or a diagnosis of type 2 diabetes or type 2 diabetes treatment;
(3) Blood pressure ≥ 130/85 mmHg or specific antihypertensive treatment;
(4) Plasma TG ≥ 1.70 mmol/L (150 mg/dL) or lipid-lowering therapy;
(5) Plasma high-density lipoprotein cholesterol (HDL-C) ≤ 1.0 mmol/L (40 mg/dL) in men and ≤ 1.3 mmol/L (50 mg/dL) in women, or lipid-lowering therapy.

### 2.6. Statistical Analysis

All primary analyses accounted for the complex NHANES survey design using the combined 6-year MEC weight (WTMEC6YR, derived from WTMEC2YR across the 2007–2008, 2009–2010, and 2011–2012 cycles), strata, and primary sampling units. The survey design object is described in Supplementary Methods.

Baseline characteristics are presented as weighted median (Q1, Q3) for continuous variables and unweighted n (weighted %) for categorical variables; group comparisons used Rao–Scott *χ*^2^ tests and weighted Wilcoxon rank-sum tests. *p* values are exploratory and were not adjusted for multiple comparisons. No imputation was performed.

Weighted multivariable logistic regression was used to estimate ORs and 95% CIs. FT3 was modeled continuously, and ORs are reported per 1-SD increase. For MASLD, Model 2 adjusted for age, gender, and race; Model 3 additionally adjusted for FT4, TSH, ALT, and AST. For high probability of advanced liver fibrosis, Model 2 adjusted for gender, race, marital status, and PIR; Model 3 additionally adjusted for TSH, FT4, TG, and GGT.

The FT4/FT3 ratio was also modeled continuously, and ORs are reported per 1-unit increase. For MASLD, Model 3 adjusted for age, gender, race, TSH, ALT, and AST; for high probability of advanced liver fibrosis, Model 3 adjusted for gender, race, marital status, PIR, TSH, TG, and GGT. An age-adjusted OR for the FT4/FT3–fibrosis association was not estimated.

Restricted cubic spline (RCS) analyses used weighted logistic regression with four knots at the 5th, 25th, 50th, and 75th percentiles and the same covariates as the corresponding fully adjusted models; P-overall and P-nonlinear were reported.

Exploratory mediation analyses used the *mediation* package (version 4.5.1) with 5000 bootstrap resamples. ALB and LYM were tested in separate models; the other mediator was not included in the outcome model. These analyses were unweighted because the mediation package cannot fully accommodate the complex NHANES survey design, and they are cross-sectional and hypothesis-generating only; causal mediation cannot be inferred. Exact covariate lists are provided in Supplementary Methods.

Sensitivity analyses used an alternative FLI cut-off (FLI ≥ 30) in the overall population and in the strict euthyroid subgroup. Strict euthyroid status was defined as TSH 0.4–4.0 mIU/L, FT3 2.0–4.4 pg/mL, and FT4 0.8–1.8 ng/dL. Because age is a mathematical component of the FIB-4 equation, age was not additionally included as a covariate in the primary FIB-4 model, to avoid overadjustment. However, residual confounding by age may remain. Therefore, we performed sensitivity analyses with additional adjustment for continuous age, age-stratified analyses (20–64 vs. ≥ 65 years), and an FT3 × age-group interaction test. The fibrosis-related results should be interpreted based on these analyses (Section 3.6 and Supplementary Table S3). ORs were expressed per 1-SD increase using weighted SDs of 0.385 pg/mL in the overall population and 0.361 pg/mL in the strict euthyroid subgroup. These analyses were exploratory.

Statistical analyses were performed using R version 4.5.2 (R Foundation for Statistical Computing, Vienna, Austria); two-sided *p* < 0.05 was considered statistically significant unless otherwise specified.

## 3. Results

### 3.1. Baseline Participant Characteristics

Table 1 presents the baseline characteristics of the participants according to MASLD diagnosis. A total of 6910 participants were included in the analysis (3184 with MASLD and 3726 without MASLD). The median participant age was 43.00 years (Q1, Q3: 31.00, 55.00). Overall, 52% were male, and 72% were non-Hispanic White.

Compared with the non-MASLD group, participants with MASLD were older (47 vs. 40, *p* < 0.001) and more likely to be male (61% vs. 44%, *p* < 0.001). The poverty income ratio (PIR) did not differ significantly between the two groups [2.81 (1.42, 4.90) vs. 2.99 (1.48, 5.00), *p* = 0.270]. In laboratory indicators, MASLD individuals had higher levels of FT3, AST, ALT, GGT, ALP and lymphocyte count than non-MASLD individuals (Table 1).

**Table 1:** Baseline characteristics of included participants stratified by MASLD diagnosis.

Characteristic	Overall N = 6910 ^1^	Non-MASLD N = 3726 ^1^	MASLD N = 3184 ^1^	*p*-Value ^2^
Age	43.00 (31.00, 55.00)	40.00 (28.00, 53.00)	47.00 (37.00, 58.00)	<0.001
Gender				<0.001
Female	3327 (48%)	2016 (56%)	1311 (39%)	
Male	3583 (52%)	1710 (44%)	1873 (61%)	
Race				<0.001
Mexican American	1203 (9.6%)	555 (8.3%)	648 (11%)	
Non-Hispanic Black	1454 (12%)	758 (12%)	696 (13%)	
Non-Hispanic White	2921 (72%)	1609 (74%)	1312 (69%)	
Other Hispanic	822 (6.4%)	434 (6.3%)	388 (6.5%)	
Marital				<0.001
Don’t Know	1 (<0.1%)	1 (<0.1%)	0 (0%)	
Married/Living with partner	4158 (64%)	2154 (61%)	2004 (68%)	
Widowed/Divorced/Separated/Never married	2751 (36%)	1571 (39%)	1180 (32%)	
Poverty income ratio, PIR	2.91 (1.45, 4.97)	2.99 (1.48, 5.00)	2.81 (1.42, 4.90)	0.270
Drinking				0.939
Yes	4698 (78%)	2492 (78%)	2206 (78%)	
No	1710 (22%)	927 (22%)	783 (22%)	
Fatty liver index, FLI	51.44 (19.29, 82.71)	22.71 (10.42, 40.36)	85.66 (73.89, 94.79)	<0.001
Fibrosis-4, FIB-4	0.84 (0.58, 1.21)	0.81 (0.56, 1.16)	0.90 (0.62, 1.25)	<0.001
Thyroid stimulating hormone, TSH (mIU/L)	1.56 (1.07, 2.32)	1.49 (1.04, 2.27)	1.66 (1.13, 2.37)	<0.001
Free thyroxine, FT4 (ng/dl)	0.80 (0.70, 0.87)	0.80 (0.70, 0.88)	0.80 (0.70, 0.85)	0.001
Free triiodothyronine, FT3 (pg/ml)	3.20 (2.98, 3.40)	3.11 (2.90, 3.40)	3.23 (3.00, 3.50)	<0.001
Serum albumin (g/L)	43.00 (41.00, 45.00)	44.00 (41.00, 46.00)	43.00 (40.00, 45.00)	<0.001
Lymphocyte count (1000 cells/uL)	2.00 (1.70, 2.50)	2.00 (1.60, 2.40)	2.20 (1.80, 2.70)	<0.001
Segmented neutrophil count (1000 cells/uL)	4.00 (3.10, 5.10)	3.80 (2.90, 4.80)	4.30 (3.40, 5.40)	<0.001
Alanine aminotransferase, ALT (U/L)	21.00 (17.00, 29.00)	19.00 (15.00, 25.00)	26.00 (20.00, 36.00)	<0.001
Aspartate aminotransferase, AST (U/L)	23.00 (20.00, 28.00)	22.00 (19.00, 26.00)	25.00 (21.00, 30.00)	<0.001
Gamma-glutamyl transferase, GGT (U/L)	20.00 (14.00, 31.00)	16.00 (12.00, 22.00)	27.00 (19.00, 41.00)	<0.001
Alkaline phosphatase, ALP (U/L)	63.00 (52.00, 77.00)	60.00 (49.00, 73.00)	68.00 (56.00, 82.00)	<0.001
Diabetes	3019 (36%)	1212 (26%)	1807 (50%)	<0.001
Hypertriglyceridemia	3114 (43%)	988 (25%)	2126 (67%)	<0.001

^1^ Continuous variables: weighted median (Q1, Q3). Categorical variables: unweighted n (weighted %). ^2^ *p* values from design-based tests (Rao–Scott χ^2^ for categorical, weighted Wilcoxon for continuous). *p* values are exploratory, not adjusted for multiple comparisons and should be interpreted with caution.

### 3.2. The Association Between FT3 and MASLD and High Probability of Advanced Liver Fibrosis

Weighted multiple logistic regression models were constructed to assess the associations of FT3 with MASLD and high probability of advanced liver fibrosis (Table 2). FT3 was modeled as a continuous variable, and ORs are presented per 1-SD increase (0.385 pg/mL). As shown in Table 2, in the fully adjusted model, FT3 was positively associated with MASLD risk (OR = 1.34, 95% CI 1.18–1.53, *p* < 0.001). Conversely, FT3 showed a significant inverse association with high probability of advanced liver fibrosis in the fully adjusted model (OR = 0.41, 95% CI 0.26–0.65, *p* < 0.001). The fibrosis-related findings should be further interpreted according to the age sensitivity analyses in Section 3.6 and Supplementary Table S3.

Model 1: Unadjusted crude model.

Model 2: Adjusted for age, gender, and race for MASLD; adjusted for gender, race, marital status, and poverty income ratio (PIR) for high probability of advanced liver fibrosis.

Model 3: Fully adjusted for age, gender, race, FT4, TSH, AST, ALT for MASLD; fully adjusted for gender, race, marital status, PIR, TSH, FT4, TG, GGT for high probability of advanced liver fibrosis.

The primary FIB-4 model did not additionally include age. Age-adjusted, age-stratified, and interaction analyses are presented in Section 3.6 and Supplementary Table S3.

### 3.3. Nonlinear Relationship Between FT3 and MASLD and High Probability of Advanced Liver Fibrosis

**Figure 2:**
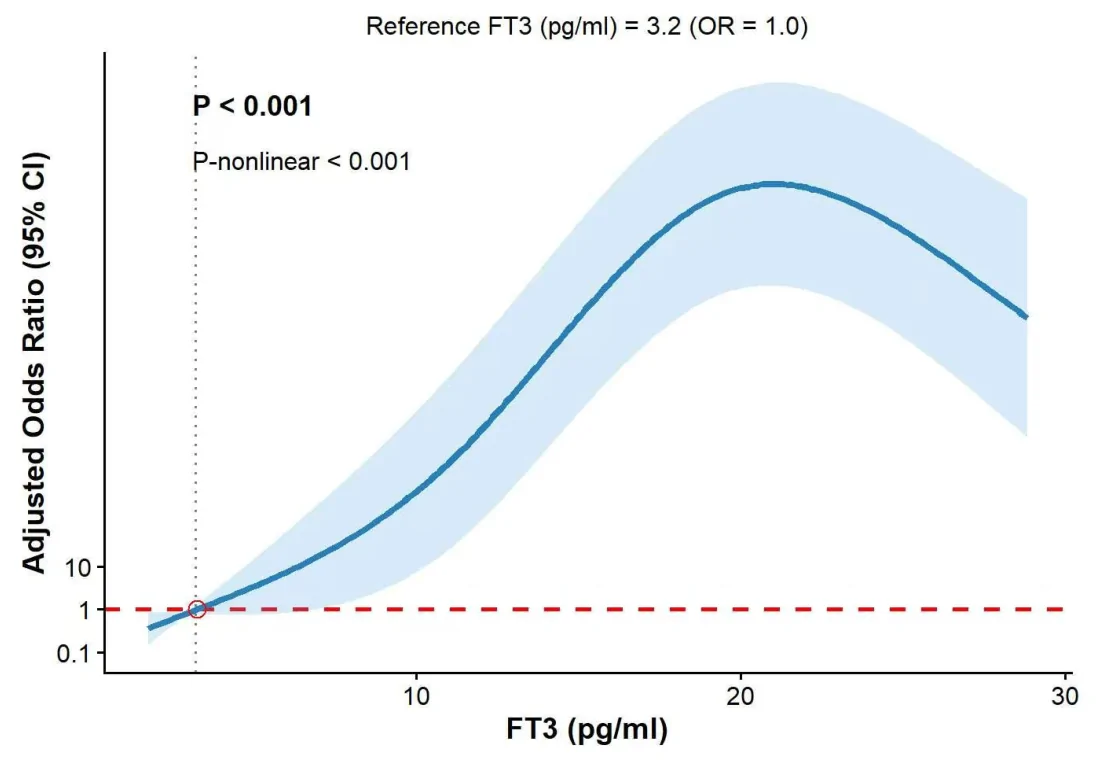
Restricted cubic spline analysis of the adjusted association between serum FT3 concentration and FLI-defined MASLD. The solid blue line shows the adjusted odds ratio; the light-blue band shows the 95% confidence interval; the red dashed line indicates OR = 1; and the dotted vertical line with an open circle marks the reference value.

**Figure 3:**
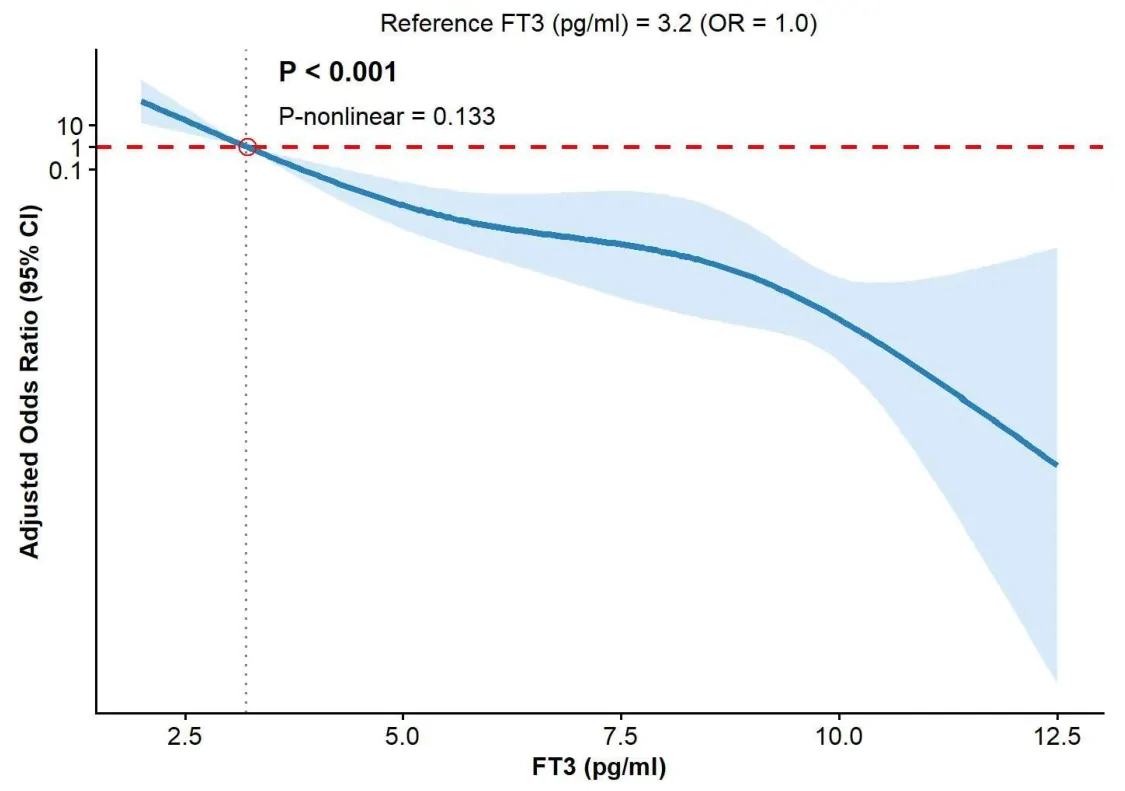
Restricted cubic spline analysis of the adjusted association between serum FT3 concentration and FIB-4-defined high probability of advanced liver fibrosis. The solid blue line shows the adjusted odds ratio; the light-blue band shows the 95% confidence interval; the red dashed line indicates OR = 1; and the dotted vertical line with an open circle marks the reference value.

We used a RCS analysis based on the weighted logistic regression in the study population to capture its potential nonlinear characteristics. For MASLD, the overall association with FT3 was statistically significant, with a notable nonlinear trend (P-overall < 0.001, P-nonlinear < 0.001) (Figure 2). In contrast, the association between FT3 and high probability of advanced liver fibrosis presented no statistically significant nonlinearity (P-nonlinear = 0.133), despite a significant overall association (P-overall < 0.001). As FT3 concentrations declined below 3.2 pg/mL, the adjusted odds ratio of high probability of advanced liver fibrosis increased steadily, demonstrating a consistent inverse linear association between FT3 and high probability of advanced liver fibrosis (Figure 3).

Model adjusted for age, gender, race, FT4, TSH, AST, ALT. Reference FT3 = 3.2 pg/mL (OR = 1.0). The solid blue line shows the adjusted odds ratio; the light-blue band shows the 95% confidence interval; the red dashed line indicates OR = 1; and the dotted vertical line with an open circle marks the reference value.

Model adjusted for gender, race, marital status, PIR, TSH, TG, GGT. Age was not additionally adjusted and these estimates are exploratory and age-dependent. Reference FT3 = 3.2 pg/mL (OR = 1.0). The solid blue line shows the adjusted odds ratio; the light-blue band shows the 95% confidence interval; the red dashed line indicates OR = 1; and the dotted vertical line with an open circle marks the reference value.

### 3.4. Relationship Between the FT4/FT3 Ratio and MASLD and High Probability of Advanced Liver Fibrosis

We further calculated the FT4/FT3 ratio and constructed weighted multivariable logistic regression models to evaluate its associations with the two hepatic outcomes (Table 3). In the present data, a higher FT4/FT3 ratio was associated with lower odds of MASLD (OR = 0.57, 95% CI: 0.42–0.77, *p* < 0.001) after age adjustment. Conversely, its positive association with a high probability of advanced liver fibrosis was derived from an age-unadjusted model (OR = 5.26, 95% CI: 2.98–9.26, *p* < 0.001). Because age was not included in this fibrosis model, the estimate carries the same limitations as the age-sensitive FT3–FIB-4 findings.

**Figure 4:**
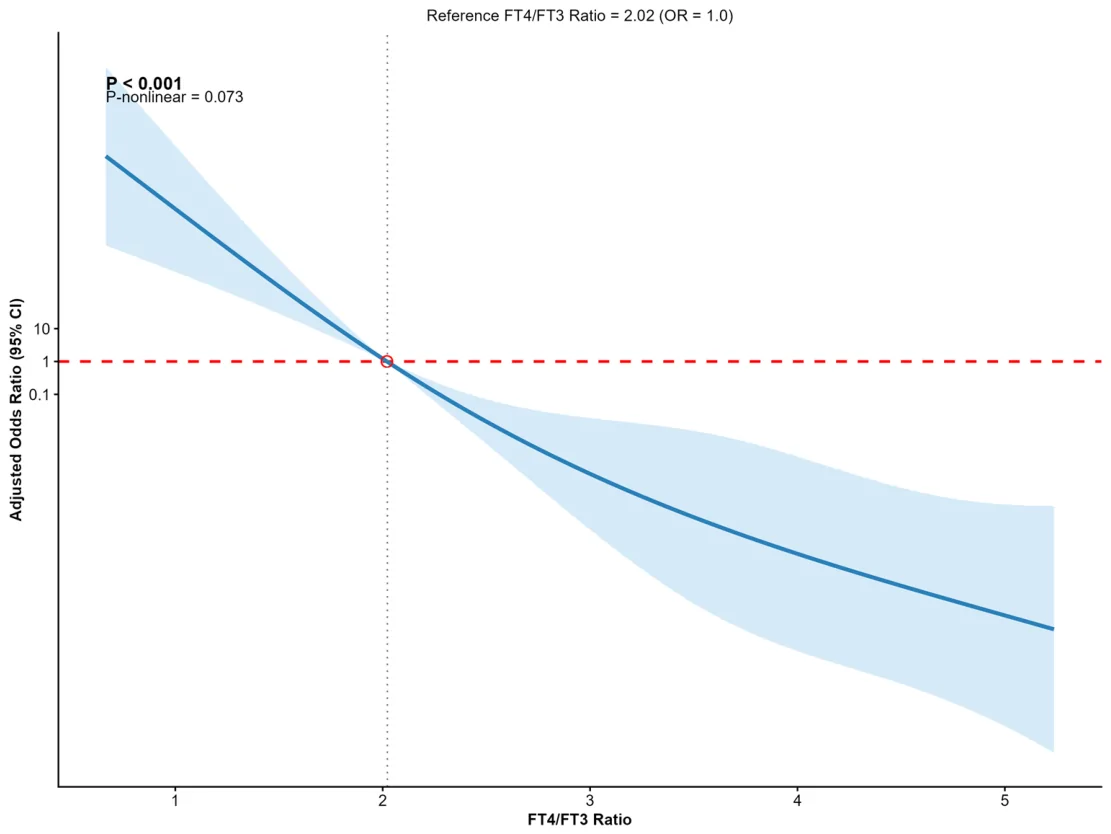
Restricted cubic spline plots for FT4/FT3 ratio and MASLD risk. An inverse linear association with MASLD risk. Shaded bands indicate 95% confidence intervals for adjusted odds ratios. The curve shows a negative linear association. The solid blue line shows the adjusted odds ratio; the light-blue band shows the 95% confidence interval; the red dashed line indicates OR = 1; and the dotted vertical line with an open circle marks the reference value.

**Figure 5:**
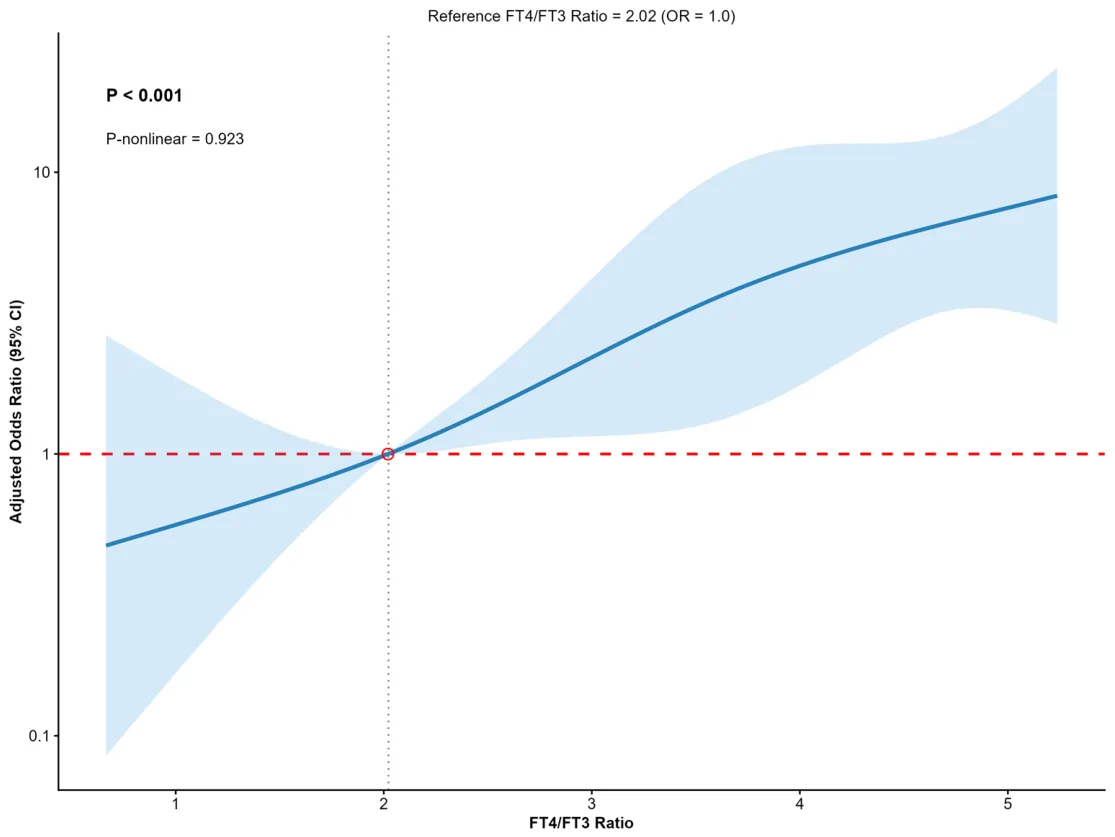
Restricted cubic spline plots for FT4/FT3 ratio and high probability of advanced liver fibrosis in the model without age adjustment. The solid blue line shows the adjusted odds ratio; the light-blue band shows the 95% confidence interval; the red dashed line indicates OR = 1; and the dotted vertical line with an open circle marks the reference value.

We subsequently performed RCS analysis to explore the relationship between the FT4/FT3 ratio and MASLD as well as high probability of advanced liver fibrosis. A linear inverse association was observed between the FT4/FT3 ratio and MASLD risk (Figure 4). In the age-unadjusted fibrosis model, a linear positive association was observed (overall *p* < 0.001; nonlinear *p* > 0.05) (Figure 5).

Model 1: Unadjusted crude model.

Model 2: Adjusted for age, gender, and race for MASLD; adjusted for gender, race, marital status, and poverty income ratio (PIR) for high probability of advanced liver fibrosis.

Model 3: Fully adjusted for age, gender, race, TSH, AST, ALT for MASLD; fully adjusted for gender, race, marital status, PIR, TSH, TG, GGT for high probability of advanced liver fibrosis.

For the FIB-4 outcome, age was not additionally included; this association is exploratory, age-dependent, and not robust.

The curve shows a negative linear association. The solid blue line shows the adjusted odds ratio; the light-blue band shows the 95% confidence interval; the red dashed line indicates OR = 1; and the dotted vertical line with an open circle marks the reference value.

The curve shows a positive linear association. Age was not additionally adjusted and these estimates are exploratory. The solid blue line shows the adjusted odds ratio; the light-blue band shows the 95% confidence interval; the red dashed line indicates OR = 1; and the dotted vertical line with an open circle marks the reference value.

### 3.5. Exploratory Mediation Analyses of the Associations Between FT3 and MASLD and High Probability of Advanced Liver Fibrosis

**Figure 6:**
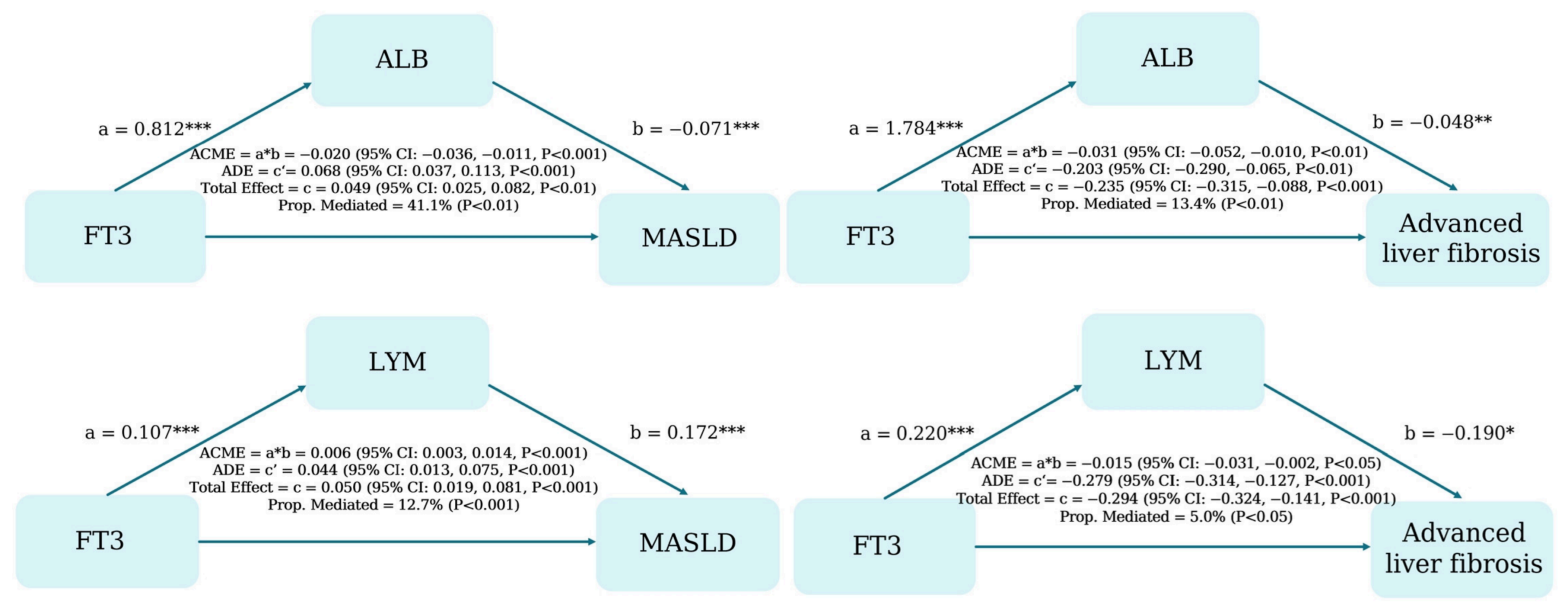
Exploratory, unweighted mediation analyses of the associations between FT3 and MASLD or a high probability of advanced liver fibrosis, examining serum albumin (ALB) and lymphocyte count (LYM). Asterisks denote statistical significance levels: *** *p* < 0.001, ** *p* < 0.01, * *p* < 0.05.

Exploratory mediation analyses with 5000 bootstrap resamples were conducted to examine whether serum albumin (ALB) and lymphocyte count (LYM) might be associated with part of the FT3–MASLD and FT3–advanced fibrosis associations. ALB and LYM were tested in separate models. As shown in Figure 6, the exploratory proportion explained by ALB was 41.1% for the FT3–MASLD association (*p* < 0.01) and 13.4% for the FT3–high probability of advanced liver fibrosis association (*p* < 0.01). The exploratory proportion explained by LYM was 12.7% for the FT3–MASLD association (*p* < 0.001) and 5.0% for the FT3–high probability of advanced liver fibrosis association (*p* < 0.05). Because the mediators were evaluated separately, these proportions are not additive and should be interpreted as the proportion explained by each mediator when considered individually.

Exploratory mediation analyses were performed with 5000 bootstrap resamples. ALB and LYM were tested in separate models.

Coefficients, average causal mediation effect (ACME, indirect effect), average direct effect (ADE), total effect, and proportion explained are shown. ACME is the indirect-effect estimate returned by the *mediation* package. These estimates are exploratory and do not establish causal mediation. Because the mediators were evaluated separately, the proportions are not additive. Asterisks denote statistical significance levels: *** *p* < 0.001, ** *p* < 0.01, * *p* < 0.05.

### 3.6. Sensitivity Analyses

In sensitivity analyses using FLI ≥ 30, the positive association between FT3 and hepatic steatosis remained directionally consistent in both the overall and strict euthyroid populations (Supplementary Table S1).

In the strict euthyroid subgroup, FT3 was positively associated with MASLD in the fully adjusted model (OR per 1 SD = 1.34, 95% CI 1.22–1.48, *p* < 0.001), whereas the association with FIB-4-defined high probability of advanced liver fibrosis was significant only in the fully adjusted model (OR per 1 SD = 0.44, 95% CI 0.24–0.73, *p* = 0.003), with only 49 events (Supplementary Table S2).

Because age is a mathematical component of FIB-4, the primary model did not additionally adjust for age. After additional adjustment for continuous age, the inverse association with FIB-4 was attenuated and no longer significant (OR per 1 SD = 0.83, 95% CI 0.50–1.37, *p* = 0.451). In age-stratified analyses, the inverse association was significant in participants aged ≥ 65 years (OR per 1 SD = 0.48, 95% CI 0.29–0.79, *p* = 0.005) but not in those aged 20–64 years (OR per 1 SD = 0.73, 95% CI 0.44–1.22, *p* = 0.222). However, the FT3 × age-group interaction was not significant (P for interaction = 0.072) (Supplementary Table S3). Therefore, the fibrosis-related finding is exploratory, age-dependent, and not robust.

## 4. Discussion

The present study examined the associations of serum FT3 with MASLD and high probability of advanced liver fibrosis among euthyroid adults. Higher FT3 was associated with increased MASLD risk. For FIB-4-defined high probability of advanced liver fibrosis, the primary model without age adjustment showed an inverse association with FT3, but after continuous age adjustment, this association attenuated and became non-significant. An inverse association was observed only in participants aged ≥65 years, with a non-significant age-group interaction. The FT4/FT3 ratio was inversely associated with MASLD in the age-adjusted model. Its positive association with a high probability of advanced liver fibrosis came from a model that did not include age and is reported as a cross-sectional association. Therefore, the fibrosis-related finding is exploratory, age-dependent, and not robust. Exploratory mediation analyses suggested that ALB and LYM might be associated with part of the FT3–MASLD association.

These findings are broadly consistent with previous evidence. Unlike previous studies that mainly focused on overt or subclinical hypothyroidism, the present study focused on FT3 variation within the euthyroid range. A prior study by Gao et al. reported that elevated FT3 levels were correlated with increased MASLD risk in euthyroid populations (OR = 1.35, 95% CI: 1.23–1.49; *p* < 0.001) [24]. Accumulating data have also demonstrated positive associations of FT3 levels with obesity, insulin resistance, and metabolic syndrome. Specifically, euthyroid obese individuals tend to present higher FT3 levels, whereas FT3 concentrations decline after weight reduction [25,26]. However, MASLD was defined using FLI, a non-invasive surrogate that incorporates triglycerides, BMI, GGT, and waist circumference; although the primary models did not adjust for these FLI components, the outcome definition may partially overlap with metabolic mediators, which should be considered when interpreting these associations.

The underlying mechanisms responsible for elevated FT3 in metabolic disorders remain incompletely elucidated. Insulin resistance, a hallmark of MASLD, may promote intracellular T3 synthesis as a compensatory response to altered hepatic metabolic homeostasis [27,28]. Enhanced local T3 production is hypothesized to restore metabolic activity and facilitate adaptive thermogenesis in metabolically impaired tissues [28,29]. In MASLD, hepatic-specific thyroid hormone resistance may further induce intrahepatic FT3 elevation, representing a tissue-specific hormonal adaptation rather than systemic thyroid dysfunction [30,31]. Collectively, these mechanistic rationalities support the credible association between FT3 variation within the normal range and MASLD progression observed in the current exploratory study.

Accumulating clinical evidence has demonstrated associations of low-normal thyroid function, subclinical hypothyroidism, and overt hypothyroidism with elevated liver fibrosis risk in general populations [25,32,33]. A previous study by Martínez-Escudé et al. reported that individuals with low-normal thyroid function (elevated TSH with normal FT3 and FT4) exhibited higher liver stiffness and a greater prevalence of hepatic fibrosis relative to those with strict-normal thyroid function [25]. The increased fibrotic risk persisted after adjusting for metabolic confounders, despite attenuated statistical significance. Focusing on FT3-specific effects, prior clinical research has verified that reduced FT3 levels are correlated with high probability of advanced liver fibrosis, indicating that adequate thyroid hormone activity may be beneficial for alleviating fibrotic progression. In the present study, the simultaneous evaluation of MASLD and high probability of advanced liver fibrosis revealed opposite association patterns with FT3, which may help refine risk stratification in euthyroid individuals. These findings should be interpreted cautiously, as detailed in the limitations.

Thyroxine (T4) and triiodothyronine (T3) constitute the major circulating thyroid hormones. T4 is converted to biologically active FT3 via type I and type II deiodinases, and FT3 serves as a sensitive indicator of peripheral thyroid function. Obesity, a well-established risk factor for metabolic-associated steatotic liver disease, can upregulate deiodinase activity. The resultant enhanced conversion of FT4 to FT3 is considered a compensatory metabolic response to mitigate excessive lipid accumulation [25,33]. Accordingly, the FT4/FT3 ratio may indirectly reflect systemic deiodinase activity and has been proposed as a candidate serological correlate in previous studies [34]; however, the present cross-sectional analysis only shows associations. In earlier metabolic disease, increased peripheral conversion of T4 to T3 may represent an adaptive response associated with obesity and insulin resistance. In more advanced liver disease, systemic illness, impaired hepatic deiodination, inflammation, and reduced hepatic synthetic function may instead be associated with lower circulating FT3. In line with previous evidence, our results showed cross-sectional associations between the FT4/FT3 ratio and both hepatic outcomes. This ratio is reported as a cross-sectional association finding, as diagnostic performance was not evaluated.

The present findings support the concept of intrahepatic hypothyroidism, which occurs independent of systemic thyroid function status. In euthyroid individuals with MASH, impaired hepatic conversion of T4 to biologically active T3, alongside enhanced production of inactive reverse T3, reduces local T3 availability. The resultant suppression of intrahepatic THRβ signaling facilitates MASH pathogenesis and fibrotic progression [12,35,36,37]. Consistent with this mechanism, resmetirom, a liver-specific thyroid hormone receptor agonist targeting THRβ, has demonstrated beneficial effects on hepatic inflammation, fibrosis, and lipid profiles in clinical trials among patients with MASH [38,39].

In exploratory analyses, ALB and LYM were separately associated with part of the FT3–MASLD association. These findings are hypothesis-generating and do not establish causal mediation. Consistent with the exploratory signal for LYM, systemic inflammation has been associated with fibrosis severity in MASLD. A cross-sectional study by Olfa et al. reported an association between the neutrophil-to-lymphocyte ratio (NLR) and significant fibrosis in MASLD patients [40]. A meta-analysis further found higher NLR levels in NAFLD patients with significant fibrosis than in those without (SMD = 1.59, 95% CI 0.76–2.43) [41]. However, evidence regarding NLR and advanced fibrosis remains inconsistent, and these findings should be regarded as exploratory. Moreover, because the study was cross-sectional, temporal ordering cannot be established, and the exploratory mediation analyses were unweighted because the *mediation* package cannot fully accommodate the complex NHANES survey design; these exploratory signals therefore require further confirmation and should not be interpreted as causal mediation.

Thyroid dysfunction also modulates hepatic oxidative status through altered antioxidant availability, which elevates reactive oxygen species accumulation and enhances systemic oxidative stress [12]. Oxidative injury and chronic inflammation are well-established drivers of MASLD progression, mediating the transition from simple steatosis to steatohepatitis and advanced fibrosis. Peripheral inflammatory indicators, including lymphocyte-based inflammatory ratios, have been consistently correlated with hepatic fibrotic severity [42]. Animal studies have further validated the protective effects of thyroid hormones against hepatic lipotoxicity, inflammation, oxidative damage, and fibrosis [43]. Mechanistically, T3 regulates hepatocytic autophagy and lysosomal activity, promoting mitochondrial fatty acid β-oxidation in a thyroid hormone receptor-dependent manner to alleviate hepatic lipid deposition [44].

Several limitations should be acknowledged. First, FLI and FIB-4 are non-invasive surrogates rather than gold-standard measurements; liver biopsy, MRI-PDFF, and vibration-controlled transient elastography were not available in current data, so misclassification of steatosis and fibrosis is possible. Second, FLI incorporates triglycerides, BMI, GGT, and waist circumference, which are closely linked to thyroid function and metabolic status; although the primary MASLD models did not adjust for these FLI components, the outcome definition may partially overlap with potential mediators. Sensitivity analyses using an alternative FLI cut-off (FLI ≥ 30) yielded directionally consistent results. Third, FIB-4 inherently includes age; the FT3–fibrosis association was sensitive to age adjustment and was based on a limited number of events. The FT4/FT3–fibrosis model likewise did not adjust for age, and an age-adjusted estimate for this ratio was not calculated. Therefore, the fibrosis-related finding should be interpreted cautiously, and future studies using age-independent fibrosis measures are warranted. Fourth, the cross-sectional design precludes temporal ordering; the exploratory mediation analyses cannot establish causality. Fifth, the exploratory mediation analyses were unweighted because the mediation package cannot fully accommodate the complex NHANES survey design, and standard errors may be underestimated. Finally, baseline age and gender differed between the MASLD and non-MASLD groups; although all models were adjusted for these covariates, residual confounding cannot be entirely excluded. Self-reported medication and alcohol data may also introduce measurement error.

## 5. Conclusions

In euthyroid adults, higher FT3 and a lower FT4/FT3 ratio were cross-sectionally associated with increased MASLD risk. Associations of lower FT3 and a higher FT4/FT3 ratio with high probability of advanced liver fibrosis were exploratory, age-dependent, and not robust. The fibrosis-related findings are cross-sectional associations and do not establish clinical utility.

## Figures and Tables

**Table 2:** Weighted multivariable logistic regression results for the association between FT3 and two hepatic outcomes (FLI-defined MASLD, FIB-4-defined high probability of advanced liver fibrosis).

Model	OR (95% CI) per 1-SD	p-Value
**MASLD**		
Model 1 (unadjusted)	1.18 (1.08, 1.28)	<0.001
Model 2 (partially adjusted)	1.26 (1.12, 1.41)	<0.001
Model 3 (fully adjusted)	1.34 (1.18, 1.53)	<0.001
**High probability of advanced liver fibrosis**		
Model 1 (unadjusted)	0.56 (0.28, 1.11)	0.094
Model 2 (partially adjusted)	0.55 (0.27, 1.15)	0.120
Model 3 (fully adjusted)	0.41 (0.26, 0.65)	<0.001

Abbreviations: OR, odds ratio; 95% CI, 95% confidence interval; SD, standard deviation. FT3 was modeled as a continuous variable. ORs are reported per 1-SD increase. The weighted SD of FT3 in the overall study population was 0.385 pg/mL and was used for all models.

**Table 3:** Weighted multivariable logistic regression analyses for associations between FT4/FT3 ratio and two hepatic outcomes.

Model	OR (95% CI)	*p*-Value
**MASLD**		
Model 1 (unadjusted)	0.61 (0.49, 0.76)	<0.001
Model 2 (partially adjusted)	0.46 (0.34, 0.60)	<0.001
Model 3 (fully adjusted)	0.57 (0.42, 0.77)	<0.001
**High probability of advanced liver fibrosis**		
Model 1 (unadjusted)	3.76 (1.93, 7.32)	<0.001
Model 2 (partially adjusted)	3.67 (1.87, 7.20)	<0.001
Model 3 (fully adjusted)	5.26 (2.98, 9.26)	<0.001

Abbreviations: OR, odds ratio; 95% CI, 95% confidence interval.

## Data Availability

The datasets used and analyzed during the current study are publicly available from the NHANES database (https://www.cdc.gov/nchs/nhanes/) (accessed on 24 October 2026). If reasonable requests are made, further data can be obtained from the corresponding author.

## Supplementary Materials

The following supporting information can be downloaded at: https://www.mdpi.com/article/10.3390/healthcare14213720/s1, Supplementary Methods S1: Survey design and weights; Supplementary Methods S2: Covariate specifications for exploratory mediation models; Table S1: Sensitivity analyses of FT3 and hepatic steatosis using FLI ≥ 30 cut-off and different populations; Table S2: Sensitivity analyses of FT3 with MASLD and high probability of advanced liver fibrosis in the strict euthyroid subgroup (N = 3561); Table S3: Age-stratified analyses of the association between FT3 and high probability of advanced liver fibrosis.
