# Supplementary material for: Association Between Free Triiodothyronine and Metabolic Dysfunction-Associated Steatotic Liver Disease in Euthyroid Population: Evidence from NHANES 2007–2012
Source: Healthcare (Basel). 2026 Nov 2;14(21):3720. doi: 10.3390/healthcare14213720 (PMC13762954; doi:10.3390/healthcare14213720)
Supplement: Supplementary file 1 [file healthcare-14-03720-s001.zip › healthcare-4554140-supplementary.pdf]

Supplementary Methods S1. Survey design and weights

The combined 6-year MEC weight (WTMEC6YR) was derived from the 2-year MEC weights (WTMEC2YR) across the 2007–2008, 2009–2010, and 2011–2012 NHANES cycles. Survey-weighted analyses used a design object constructed with the R survey package as follows: *svydesign(id = ~SDMVPSU, strata = ~SDMVSTRA, weights = ~WTMEC6YR, nest = TRUE).*
In this object, SDMVPSU, SDMVSTRA, and WTMEC6YR represent the primary sampling units, strata, and the combined 6-year MEC weight, respectively.

Supplementary Methods S2. Covariate specifications for exploratory mediation models

Exploratory mediation analyses were performed using the mediation package with 5,000 bootstrap resamples. Because the mediation package cannot fully accommodate the complex NHANES survey design, these analyses were unweighted, and they should be interpreted as exploratory and hypothesis-generating only; causal mediation cannot be inferred from this cross-sectional design.

Serum albumin (ALB) and lymphocyte count (LYM) were tested in separate models. The other mediator was not included in the outcome model to avoid overadjustment. Mediator and outcome models were refitted on their common observations to ensure identical analytic samples.

For the MASLD outcome, the ALB mediator model included FT3 and FT4, and the corresponding outcome model included FT3, ALB, and FT4. The LYM mediator model included FT3 and FT4, and the corresponding outcome model included FT3, LYM, FT4, and TSH.

For the FIB-4-defined high probability of advanced liver fibrosis outcome, the ALB mediator model included FT3, FT4, and TSH, and the corresponding outcome model included FT3, ALB, FT4, and TSH. The LYM mediator model included FT3, GGT, race, gender, FT4, and TSH, and the corresponding outcome model included FT3, LYM, GGT, race, gender, FT4, and TSH.

Supplementary Table S1. Sensitivity analyses of FT3 and hepatic steatosis using FLI ≥ 30 cut-off and different populations.

Model	OR per 1 SD (95% CI)	P-value
**FLI ≥ 30 cut-off in the overall population (N= 6910)**		
**Model 1**	1.17 (1.07–1.28)	<0.001
**Model 2**	1.25 (1.10–1.43)	0.001
**Model 3**	1.18 (1.04–1.33)	0.010
**FLI ≥ 30 cut-off in the strict euthyroid subgroup (N= 3561)**		
**Model 1**	1.25 (1.14–1.37)	<0.001
**Model 2**	1.46 (1.29–1.66)	<0.001
**Model 3**	1.33 (1.17–1.52)	<0.001

Abbreviations: OR, odds ratio; 95% CI, 95% confidence interval; SD, standard deviation.

Model 1 was unadjusted. Model 2 was adjusted for age, gender, and race. Model 3 was further adjusted for FT4, TSH, ALT, and AST.

FLI ≥ 30 was present in 4,737 of 6,910 participants in the overall population and in 2,377 of 3,561 participants in the strict euthyroid subgroup.

FT3 was modeled as a continuous variable. ORs are reported per 1-SD increase. The weighted SD of FT3 was 0.385 pg/mL in the overall population and 0.361 pg/mL in the strict euthyroid subgroup; the corresponding SD was used for each analysis.

P values were derived from Wald tests for the FT3 coefficient in survey-weighted quasibinomial models.

The strict euthyroid subgroup was defined as TSH 0.4–4.0 mIU/L, FT3 2.0–4.4 pg/mL, and FT4 0.8–1.8 ng/dL.

Supplementary Table S2. Sensitivity analyses of FT3 with MASLD and high probability of advanced liver fibrosis in the strict euthyroid subgroup (N = 3561).

**Model**	**OR per 1 SD (95% CI)**	**P-value**
**MASLD**		
Model 1	1.25 (1.17–1.35)	<0.001
Model 2	1.45 (1.32–1.59)	<0.001
Model 3	1.34 (1.22–1.48)	<0.001
**High probability of advanced liver fibrosis**		
Model 1	0.58 (0.24–1.30)	0.178
Model 2	0.63 (0.24–1.64)	0.338
Model 3	0.44 (0.24–0.73)	0.003

Abbreviations: OR, odds ratio; 95% CI, 95% confidence interval; SD, standard deviation.
FT3 was modeled as a continuous variable. ORs are reported per 1-SD increase. The weighted SD of FT3 in the strict euthyroid subgroup was 0.361 pg/mL and was used for all models. P values were derived from Wald tests in survey-weighted quasibinomial models.
Model 1 was unadjusted.
For MASLD, Model 2 was adjusted for age, gender, and race; Model 3 was further adjusted for FT4, TSH, ALT, and AST.
For high probability of advanced liver fibrosis, Model 2 was adjusted for gender, race, marital status, and PIR; Model 3 was further adjusted for TSH, FT4, TG, and GGT.
The strict euthyroid subgroup was defined as TSH 0.4–4.0 mIU/L, FT3 2.0–4.4 pg/mL, and FT4 0.8–1.8 ng/dL.
In the strict euthyroid subgroup, there were 3,561 participants, of whom 1,589 had MASLD and 49 had a high probability of advanced liver fibrosis. The number of FIB-4 events in some strata was small; therefore, these results should be interpreted with caution.

Supplementary Table S3. Age-stratified analyses of the association between FT3 and high probability of advanced liver fibrosis

Model	OR per 1 SD (95% CI)	P-value
The continuous age sensitivity model	0.83 (0.50–1.37)	0.451
**Age-strata**		
20-64	0.73 (0.44–1.22)	0.222
≥65	0.48 (0.29–0.79)	0.005
**FT3 × age-group interaction**	—	0.072

Abbreviations: OR, odds ratio; 95% CI, 95% confidence interval; SD, standard deviation.

FT3 was modeled as a continuous variable. ORs are reported per 1-SD increase. The weighted SD of FT3 was 0.385 pg/mL and was used for all models. P values were derived from Wald tests for the FT3 coefficient in survey-weighted quasibinomial models. All models were adjusted for gender, race, marital status, PIR, TSH, FT4, TG, and GGT; the continuous age sensitivity model was further adjusted for age. Age-stratified analyses were performed for participants aged 20–64 years and ≥65 years.

In the overall population, 6,910 participants were included, of whom 3,184 had MASLD and 94 had a high probability of advanced liver fibrosis (FIB-4 > 2.67). The number of FIB-4 events in some strata was small; therefore, these results should be interpreted with caution.
